# Realising the potential of linked data in healthcare performance assessment efforts in New South Wales, Australia

**DOI:** 10.23889/ijpds.v1i1.310

**Published:** 2017-04-19

**Authors:** Kim Sutherland, Christopher Papadopoulos, Sadaf Marashi-Pour, Huei-Yang Chen, Jean-Fr'ed'eric L'evesque

**Affiliations:** Bureau of Health Information; Centre for Primary Health Care and Equity, University of New South Wales

## Objectives

To describe: (1) a stepwise guide to the use of linked data in the development of individual healthcare performance metrics; and (2) the application of these metrics in comprehensive performance assessment efforts at a system, regional and hospital level of analysis.

## Approach

A stepwise guide to the definition and validation of linked-data based indicators was developed following a rapid review of peer reviewed and grey literature and a series of semi-structured interviews with international subject experts. The review and interviews adopted a snowball approach to collect information on the ways in which linked data are currently used in performance measurement efforts. Data collection continued until indicator type saturation was reached. Details about existing applications of linked data in performance reporting were mapped, and used to inform the development of the guide. Indicators were aligned within a conceptual framework that is used to assess healthcare performance in New South Wales, on the basis of accessibility, appropriateness, effectiveness, efficiency, equity and sustainability.

## Results

The guide to indicator development considers the use and contribution of linked data in four stages: defining a cohort; capturing outcomes of interest; risk adjustment; and attribution. A series of vignettes illustrate the various contributions that linked data can make to performance measurement efforts - highlighting the ways in which those data can enhance understanding of complexity and dynamic relationships, and help build a comprehensive picture of performance.

## Conclusion

In performance measurement efforts internationally, linked data are used to strengthen the reliability, accuracy and precision of individual metrics and to inform efforts to assess various dimensions of healthcare performance. In New South Wales, linked data underpin publicly reported performance measures and have the potential to provide in the future whole-of-government and whole-of-system perspectives on health.

